# Estimating Causal Effects of Local Air Pollution on Daily Deaths: Effect of Low Levels

**DOI:** 10.1289/EHP232

**Published:** 2016-05-20

**Authors:** Joel Schwartz, Marie-Abele Bind, Petros Koutrakis

**Affiliations:** Department of Environmental Health, Harvard T.H. Chan School of Public Health, Boston, Massachusetts, USA

## Abstract

**Background:**

Although many time-series studies have established associations of daily pollution variations with daily deaths, there are fewer at low concentrations, or focused on locally generated pollution, which is becoming more important as regulations reduce regional transport. Causal modeling approaches are also lacking.

**Objective:**

We used causal modeling to estimate the impact of local air pollution on mortality at low concentrations.

**Methods:**

Using an instrumental variable approach, we developed an instrument for variations in local pollution concentrations that is unlikely to be correlated with other causes of death, and examined its association with daily deaths in the Boston, Massachusetts, area. We combined height of the planetary boundary layer and wind speed, which affect concentrations of local emissions, to develop the instrument for particulate matter ≤ 2.5 μm (PM2.5), black carbon (BC), or nitrogen dioxide (NO2) variations that were independent of year, month, and temperature. We also used Granger causality to assess whether omitted variable confounding existed.

**Results:**

We estimated that an interquartile range increase in the instrument for local PM2.5 was associated with a 0.90% increase in daily deaths (95% CI: 0.25, 1.56). A similar result was found for BC, and a weaker association with NO2. The Granger test found no evidence of omitted variable confounding for the instrument. A separate test confirmed the instrument was not associated with mortality independent of pollution. Furthermore, the association remained when all days with PM2.5 concentrations > 30 μg/m3 were excluded from the analysis (0.84% increase in daily deaths; 95% CI: 0.19, 1.50).

**Conclusions:**

We conclude that there is a causal association of local air pollution with daily deaths at concentrations below U.S. EPA standards. The estimated attributable risk in Boston exceeded 1,800 deaths during the study period, indicating that important public health benefits can follow from further control efforts.

**Citation:**

Schwartz J, Bind MA, Koutrakis P. 2017. Estimating causal effects of local air pollution on daily deaths: effect of low levels. Environ Health Perspect 125:23–29; http://dx.doi.org/10.1289/EHP232

## Introduction

Starting in the late 1980s, a large literature of time series studies have reported associations of daily air pollution concentrations with daily deaths (Analitis et al. 2006; Bell et al. 2004, 2013; Carbajal-Arroyo et al. 2011; Dominici et al. 2005; Fischer et al. 2003; Krall et al. 2013; Maynard et al. 2007; Peng et al. 2013; Samoli et al. 2006; Schwartz 2004a, 2004b; Stölzel et al. 2007; Zanobetti and Schwartz 2008, 2009). The most consistent results have been that particle concentrations are associated with daily mortality.

Fewer studies have examined the effects of source-specific particle contributions or individual particle species. Several large multicity studies have reported stronger associations for particle sulfate and nickel (Bell et al. 2014; Dai et al. 2014; Franklin et al. 2008). The U.S. Environmental Protection Agency’s (EPA’s) recent transport regulation has already produced substantial reductions in sulfate particles, and is scheduled to reduce remaining sulfur emissions further in the next few years (U.S. EPA 2016). As the sulfate contribution to particle mass declines and NO_x_ (nitrogen oxides) controls affect secondary organic particle formation, local emissions of particulate and gaseous pollutants will become a more important part of the pollution mix; thus it is important to enhance our understanding of their health impact.

The observational epidemiology studies cited above have been associational studies, which do not assess causality. In general, when arguing for the causality of observed associations, authors have relied on Hill’s Criteria (Hill 1965). For example, Brook et al. (2010) state “Many potential biological mechanisms exist whereby PM exposure could exacerbate existing CVDs [cardiovascular diseases] and trigger acute cardiovascular events (over the short term) and instigate or accelerate chronic CVDs (over the long run).” Besides biological plausibility, the PM_2.5_ (particulate matter ≤ 2.5 μm) epidemiological studies were relatively consistent, and exposure preceded effect.

The strength of the biological plausibility argument has grown over time (Brook et al. 2004), and includes studies indicating that particle exposure can induce lung and systemic inflammation (Adamkiewicz et al. 2004; Adar et al. 2007a; Araujo 2010; Brook 2008; Driscoll 2000; Dye et al. 2001; Folkmann et al. 2007), increase blood pressure (Baccarelli et al. 2011; Bartoli et al. 2009; Brook et al. 2009; Hoffmann et al. 2012; Schwartz et al. 2012; Wilker et al. 2010; Zanobetti et al. 2014), impair microvascular function (Brauner et al. 2008), increase coagulation and thrombosis (Baccarelli et al. 2007, 2008; Bind et al. 2012; Bonzini et al. 2010; Carlsten et al. 2007; Chuang et al. 2007; Gilmour et al. 2005; Nemmar et al. 2002), produce autonomic changes (Adar et al. 2007b; Chahine et al. 2007; Chan et al. 2004; Ghelfi et al. 2008; Zhong et al. 2015), accelerate atherosclerosis (Adar et al. 2010; Allen et al. 2009; Araujo et al. 2008; Bauer et al. 2010; Bhatnagar 2006; Hansen et al. 2007; Hoffmann et al. 2007; Sun et al. 2005, 2008; Suwa et al. 2002; Tzeng et al. 2007), and destabilize atherosclerotic plaque (Suwa et al. 2002).

There are fewer and less consistent studies assessing the effects of particle components. For example, Krall et al. (2013) and Bell et al. (2014) reported a greater toxicity for elemental (or black) carbon, a large fraction of which is associated with local traffic and domestic heating, whereas Franklin et al. (2008), Beelen et al. (2015) and Dai et al. (2014) found greater effects for sulfur and not elemental carbon.

There is biological support for a role of local traffic particles. Diesel particles have been shown to increase oxidative stress in endothelial cells (Furuyama et al. 2006; Hirano et al. 2003), inducing the production of heme oxygenase-1, a rapid response part of the body’s defense system against oxidative stress (Choi and Alam 1996). The viability of cell cultures of microvascular endothelial cells was also impaired by diesel particles with an accompanying large increase in induction of heme oxygenase-1 (Hirano et al. 2003).

A key gap in the analysis of the acute effects of local air pollution sources has been the lack of studies done in the framework of causal modeling, specifying potential outcomes, and basing their analysis on estimating the difference or ratio of potential outcomes under different exposures. In this paper, we use a causal modeling framework to estimate the causal acute effects of local pollution on daily deaths.

## Methods

### Causal Modeling

To establish causality specification of potential outcomes is required. We designate *Y_i_^A = a^* as the outcome that would occur given an exposure *A = a* for the unit *i*, and *Y_i_^A = a^* ^´^ to be the outcome that would occur if the unit *i* were instead exposed to an alternative exposure, A = a´. Causal modeling seeks to estimate the ratio of the expected value of outcome in the population of subjects *i* under the exposure they received versus what it would have been had they received the alternative exposure: *E*(*Y_i_^A = a^*)/*E*(*Y_i_^A =^* ^a´^). Because only one potential outcome is observed, various methods seek legitimate surrogates for the unobserved potential outcome (Hernán et al. 2008). In this paper, we apply the approach of instrumental variables. An instrumental variable is a variable that is related to outcome only through the exposure of interest.

### Instrumental Variables

**Figure 1:**
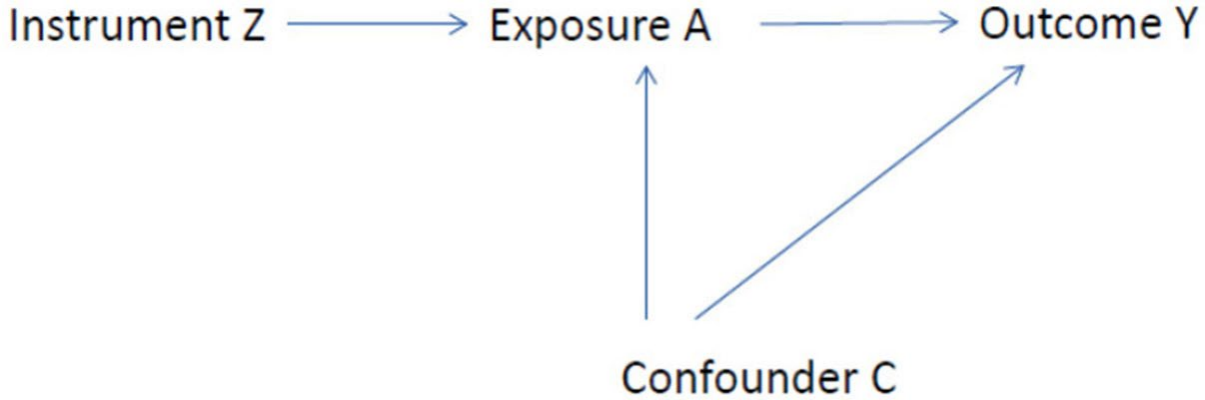
Directed acyclic graph illustrating an instrumental variable *Z*. The association between *Z* and *Y* is not confounded by *C*. By calibrating the instrument to *A*, estimates of causal effects of increases in *A* can be obtained.

Let *Y_t_^A = a^* be the potential outcome (total deaths) in the population of a city exposed to *A = a* on day *t*, and let *Y_t_^A = a^* ^´^ be the potential outcome under the alternative exposure *a*´. We would like to estimate *E*(*Y_t_^A = a^*)/*E*(*Y_t_^A = a^*´), but only *Y_t_^A = a^* is observed. We assume the potential outcome depends on predictors as follows:

Log[*E*(*Y_t_^A^* ^=^ *^a^*)] = θ_0_ + *a*θ_1_ + Φ*_t_*, [1]

where Y_t_^A = a^ represents the potential outcome at time *t* under exposure *a*, θ_0_ and θ_1_ are the intercept and the slope of exposure, respectively, and Φ*_t_* represents all of the other predictors of outcome. Unless we have measured all of the confounders, standard methods, including standard approaches to causal modeling, will give biased estimates of θ_1_. However, air pollution has many sources of variation. If there is a variable *Z* that is one such source of variation in exposure, and *Z* is associated with *Y* only through *A*, then *Z* is called an instrumental variable. Figure 1 shows the directed acyclic graph (DAG) for this scenario. Consequently, *A_t_* can be expressed as follows:

*A_t_* = *Z_t_*δ + η*_t_*, [2]

where η*_t_* represents the other sources of variation in exposure, and particularly all of the exposure variations that are associated with other measured or unmeasured predictors of outcome. This follows because of the instrument assumption, that *Z* is only related to *Y* through *A*. Formally, *E*(*Z_t_*Φ*_t_*) = 0 because of the instrument assumption. Then let *Z1* and *Z2* be equal to *Z* such that:

*E*(*A*|*Z1*) = *a*, and *E*(*A*|*Z2*) = *a*´.

Consequently,

Log[*E*(*Y_t_^Z = Z1^*)] = *E*(θ_0_ + θ_1_ *a* +Φ*_t_* |*Z* = *Z1*) = θ_0_ + θ_1_ *a* + *E*(Φ*_t_*) [3]

and

log[*E*(*Y_t_^Z = Z2^*)] = *E*(θ_0_ + θ_1_ *a*´ + Φ_t_|*Z* = *Z2*) = θ_0_ + θ_1_ *a*´ + *E*(Φ*_t_*) [4]

therefore

log[*E*(*Y_t_^Z = Z1^*)] – log[*E*(*Y_t_^Z = Z2^*)] = θ(*a* – *a*´). [5]

As a result, if we use *Z* as an instrument for *A*, we can recover a causal estimate for θ, which is the log rate ratio. Importantly, this is true even if there are unmeasured confounders.

Put less formally, in an observational study the exposure is not randomly assigned, so it may be correlated with other predictors of the outcome. However, air pollution (and other exposures) varies for many reasons. Some of them may be correlated with other predictors of daily deaths. For example, worse-than-average traffic on 1 day will increase both air pollution and stress. However, some sources of variation in air pollution may not be correlated with other predictors of daily deaths. For example, wind speed is unlikely to be correlated with daily stress, smoking, and the like. Hence, if this is true, the fraction of air pollution variation that is produced by wind speed is randomized with respect to confounders, including unmeasured ones; and if that fraction is associated with daily deaths, the estimated effect should be causal. We discuss this further below.

### Planetary Boundary Layer and Wind Speed as Instruments

The difficulty with instrumental variable analyses is finding a valid instrument that is associated only with outcome through the exposure of interest. Mendelian randomization is an example of an instrumental variable successfully applied in epidemiology, and is justified by knowledge that the biological pathway by which the genotype is associated with exposure is not associated with other predictors of outcome (Holmes et al. 2014). Hence external knowledge is critical to the technique.

The air pollution above a city is a mix of locally emitted pollutants and pollutants transported from elsewhere. The lowest part of the atmosphere, along with its behavior, is influenced by its contact with a planetary surface, which is called planetary boundary layer (PBL) and is characterized by strong vertical mixing (Finlayson-Pitts and Pitts 1986). Above the PBL lies the free atmosphere, which is mostly nonturbulent. The transport of pollutants from the boundary layer to the free atmosphere is slow relative to their vertical mixing within the boundary layer (Seinfeld and Pandis 1998). Therefore, the impact of local emissions on pollutant levels is directly related to the height of the PBL (e.g., for the same local emissions, concentrations of locally emitted pollutants are higher when the boundary layer is low and vice versa) (Seinfeld and Pandis 1998). As a result, the influence of the local emissions is modified by the atmospheric conditions. Over land, the PBL height exhibits a strong diurnal variability, with lower values at night. In addition, the mean PBL height varies substantially from day to day (Seinfeld and Pandis 1998). Besides the vertical transport (influenced by the PBL), locally emitted air pollutants are also transported horizontally, where the influence of local sources increases with decreasing wind speed and vice versa. It is hard to imagine how the PBL height can be directly related to health except through air pollution. Similarly, outside of extreme events, wind speed is an unlikely predictor of health other than through air pollution. As such, PBL height and wind speed represent attractive options as instruments for local pollution. However, PBL height and wind speed may vary seasonally and with temperature and other meteorological parameters. We believe that within strata of month and deciles of temperature, further association with predictors of health is unlikely. Hence we looked at local air pollution variation only within month-by-year strata and within deciles of temperature (for the full period), and calibrated that variation with our instruments—that is, we assume short-term predictors of mortality such as smoking, anger, and the like to be uncorrelated with PBL height on a day-to-day level, within month-by-year and decile of temperature. Our analysis took this into account.

A low PBL height and low wind speed are associated with increases in the concentrations of all locally emitted pollutants. Hence, when combined into an instrument, it can tell us that local pollution increases mortality rates (or not), but it will be difficult to identify which pollutants are responsible for the changed mortality rate.

If a single variable is used as an instrument, that variable can obtain the estimated causal effect of exposure on the outcome by regressing the outcome on the instrument, and the instrument on the exposure of interest. The product of those coefficients is the estimated causal effect per unit increase in exposure. Because we have four instrumental variables (PBL and wind speed at lag 0 and lag 1), we regressed the pollution against the four variables first, and used that result (the variation in pollution explained by the four instrumental variables) to generate a single instrumental variable for regression on the outcome. We have chosen to use these variables as instruments for PM_2.5_ (particulate matter with aerodynamic diameter ≤ 2.5 μm) as the pollutant most strongly associated with daily deaths. However, this does not demonstrate that the results are attributable exclusively to particles. We evaluated two alternative air pollutant exposures as a sensitivity analysis: black carbon (BC), which represents traffic particles, a large fraction of them locally emitted, and nitrogen dioxide (NO_2_), which is mostly from local combustion.

## Data

### Mortality Data

We analyzed data from the Boston metropolitan area, which includes the following counties: Middlesex, Norfolk, and Suffolk. Mortality data were obtained from the Massachusetts Department of Public Health for the years 2000–2009. The mortality files provided information on the exact date of death and the underlying cause of death. We chose all-cause non-accidental daily mortality [*International Classification of Diseases, 9th Revision* (ICD-9) codes 0–799] as our outcome to ensure sufficient statistical power.

### Air Quality Data

PM_2.5_ and BC measurements were conducted at the Harvard Supersite located on the roof of the Countway Library of the Harvard Medical School near downtown Boston. Ambient BC was measured continuously using an aethalometer (Magee Scientific), and PM_2.5_ was measured continuously using a tapered element oscillating microbalance (model 1400a; Rupprecht & Pataschnick Co). Daily averages were computed from the hourly values. We used publicly available daily data on the height of the PBL obtained from the NOAA (National Oceanic and Atmospheric Administration) Reanalysis Data (NOAA 2010). Ambient temperature and wind speed were obtained from the Logan Airport meteorological station.

### Analysis

First we orthogonalized our local air pollution exposures to season and temperature by fitting them to a model with dummy variables for each month of each year, and for each decile of temperature. We used four individual variables to derive one single pollution-calibrated instrumental variable: PBL height and wind speed on the day of death (lag 0) and PBL height and wind speed on the day before death (lag 1). To do this, we used a support vector regression (SVM) (Cortes and Vapnik 1995) with a radial kernel to estimate the remaining variation in PM_2.5_ (or in BC or NO_2_) that was explained by those four variables and their products including potential nonlinear dependencies on the predictors. This approach (support vector kernel regression with the radial basis kernel) combines our four instruments into one pollution calibrated instrument, and allows us to compare interquartile range (IQR) changes in the instruments for local pollution computed using each of the pollutants (PM_2.5_, BC, or NO_2_) as an indicator. The kernel regression also incorporates a ridge penalty to shrink the coefficients of the multiple terms to avoid overfitting and collinearity problems. We chose the parameters of the SVM to maximize 10-fold cross-validated *R* ^2^. We used the svm function in the R package *e1071* (version 3.2; R Project for Statistical Computing). We checked the *R* ^2^ of the instrument predicting exposure to ensure our instrument was not too weakly associated with exposure to detect an effect. Because previous literature has most commonly used the mean of PM_2.5_ on the day of death and the day preceding death as the exposure of interest, we used the mean of the instrumental variable on the day of death and the day preceding the death as our exposure, and fit a quasi-Poisson regression (allowing for overdispersion) predicting all-cause mortality. We stratified by each month of each year and by deciles of temperature, using indicator variables, and estimated the rate ratio for the instrument.

Boston has lower than average pollution levels for a U.S. city, and there were no violations of the NO_2_ annual National Ambient Air Quality (https://www.epa.gov/criteria-air-pollutants/naaqs-table) standard of 53 ppb during the study period. There were 19 days which exceeded the new U.S. EPA PM_2.5_ daily standard of 35 μg/m^3^. To assure our results apply to low-dose exposures, we repeated the analyses with the instrument excluding days when PM_2.5_ exceeded 30 μg/m^3^ to ensure that even with measurement error the exposure was below the ambient standard. This excluded 39 days. There are currently no standards for BC.

**Figure 2:**
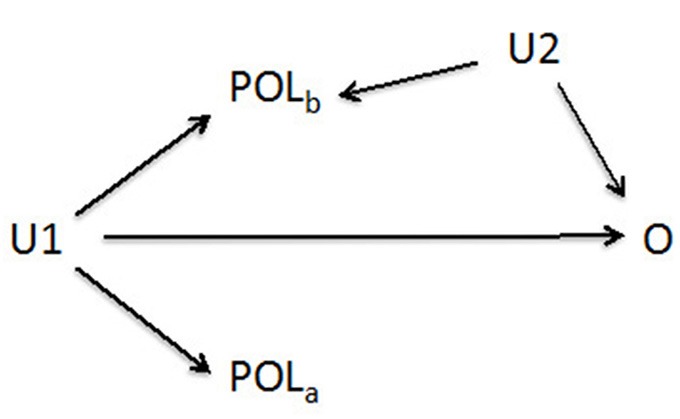
Directed acyclic graph for the Granger causality model. Confounder *U2* is measured and controlled, but confounder *U1* is not. *POL_b_* is pollution before the outcome (*O*), and *POL_a_* is pollution after the outcome. If *U1* is not controlled, there is a backdoor path from *O* to *POL_a_*, and an association would be expected. Hence, failure to find an association is evidence of a lack of confounding (i.e., no *U1*).

Granger causality is not a true causal modeling approach, but a heuristic one that argues that omitted covariates that are correlated with time-varying exposure and outcome are as likely to be correlated with tomorrow’s exposure as with yesterday’s exposure. Hence, if no association is found between future values of exposure and outcome, that suggests there is no omitted confounder. Flanders et al. (2011) give a stronger causal framework using DAGs, and note that the Granger causality approach assumes that, conditional on exposure and all confounders, exposure after the outcome should be uncorrelated with the outcome. However, exposure after the outcome and exposure before the outcome are both associated with the confounders, as illustrated in the DAG in Figure 2. Therefore, in the presence of omitted confounders an association may be expected with the future exposure. Hence, if we fit a model with the past exposure and the future exposure and find an association only with the past exposure, that would argue against such omitted confounders, and vice versa. We tested this approach by rerunning our instrumental variable model with the mean of the instrument (lags 0 and 1) and the mean value of the instrument on the second and third days after death. We left 1 day between the exposure before the event and the exposure after the event to produce more stable estimates for each association, given the serial correlation in pollution.

We also conducted a sensitivity analysis to test our assumption that we had a valid instrument. Looking at Figure 1 again, we see that the instrumental variable (*Z*) is associated only with the outcome through the exposure (*A*) (the assumption for instrumental variables). That is, the exposure can be viewed as a mediator of the association of the instrumental variable with the outcome. Then if we control for *A*, there should be no association with the instrument any longer (no direct effect) by that assumption. If, in contrast, an association remains, then there is another path from *Z* to the outcome, through some confounder. We tested this by fitting a model with both our instrument and the original exposure variable (PM_2.5_).

To put our results in context, we performed a quantitative health impact assessment. Specifically, we estimated the reduction in deaths during the 10 years of study for an IQR reduction in our instrumental variable (after ensuring that such a reduction from the mean would result in an exposure above zero). This was estimated as


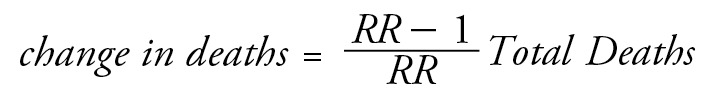


where *RR* is the rate ratio for the change in exposure, exp(b1 × IQR) where b1 is the coefficient of the instrumental variable, and IQR is its interquartile range. This approach is standard in risk assessment (Fann et al. 2011; GBD 2013 Risk Factors Collaborators et al. 2015; U.S. EPA 1999). We computed the total deaths during follow-up (204,386) from our data.

## Results

Table 1 shows descriptive statistics for the variables in our study. Air pollution concentrations were low, and almost always well below the current U.S. EPA standards (results not shown). Table 2 shows the correlations among the covariates. The correlation between PM_2.5_ and BC was 0.65, between PM_2.5_ and NO_2_ was 0.45, and between BC and NO_2_ was 0.57. The correlation between air pollution and the candidate instruments were modest. For example, for PM_2.5_, the correlation with PBL height was –0.35, and with wind speed was –0.28.

**Table 1:** Descriptive statistics of the data: air pollution and daily deaths in Boston, 2000–2009.

Variable	Mean ± SD	Min	Max
Daily deaths^*a*^	55.8 ± 9.5	27	94
PM_2.5_ (μg/m^3^)^*b*^	9.8 ± 5.8	0.2	67.2
BC (μg/m^3^)^*b*^	0.70 ± 0.41	0.10	4.70
NO_2_ (ppb)^*c*^	18.4 ± 6.4	4.0	46.9
PBL (m)^*d*^	770 ± 356	110	2,392
Temperature (°C)^*e*^	10.8 ± 9.4	–16.9	31.5
Wind speed (knots)^*e*^	9.6 ± 3.2	2.5	26
Abbreviations: max, maximum; min, minimum. ^***a***^Data from MA Department of Public Health. ^***b***^Data measured at Harvard Supersite. ^***c***^Data from MA Department of Environmental Protection. ^***d***^Data from NOAA North America Reanalysis data set (NOAA 2010). ^***e***^Data from National Climatic Data Center.			

**Table 2:** Correlation matrix of the exposures.

	PM_2.5_ (μg/m^3^)	BC (μg/m^3^)	NO_2_ (ppb)	PBL (m)	Temperature (°C)	Wind speed (knots)
PM_2.5_ (μg/m^3^)	1
BC (μg/m^3^)	0.65	1
NO_2_ (ppb)	0.45	0.57	1
PBL (m)	–0.35	–0.52	–0.35	1
Temperature (°C)	0.30	0.26	–0.25	–0.23	1
Wind speed (knots)	–0.28	–0.52	–0.37	0.54	–0.28	1

### Instrumental Variable Model

If a model predicting a variable is over fit (e.g., uses too many degrees of freedom), then one would expect the predicted *R* ^2^ on left-out monitors to be noticeably smaller than the model *R* ^2^ in the training data set. The cross-validated *R* ^2^ of the instrumental variable predicting PM_2.5_ was 0.180, little changed from the *R* ^2^ in the training data (0.189). Although low, this is consistent with the fact that most of the PM_2.5_ in Boston is transported rather than locally emitted, and with PM having other important sources of variation besides PBL and wind speed (Masri et al. 2015). Overfitting was avoided because the tuning parameters of the model calibrating the instrument to PM_2.5_ were chosen by cross-validation, and because the SVM uses a ridge penalty, where a penalty term is added to the cost function proportional to the sum of the square of the regression coefficients. This penalty constrains the coefficients from varying wildly, or growing too large.

As expected, PBL height and wind speed were better predictors of BC (a large fraction of which is locally emitted) than of PM_2.5_. The cross-validated *R* ^2^ of the SVM model for BC was 0.36, versus 0.37 without cross-validation. Similarly, the SVM model for NO_2_ had a cross-validated *R* ^2^ of 0.39, versus 0.40 without cross-validation.

### Mortality Model

An IQR change in the instrument for local PM_2.5_ was associated with a 0.90% increase in daily deaths [95% confidence interval (CI): 0.25, 1.56], whereas an IQR change in the instrument for BC was associated with a 0.90% increase in daily deaths (95% CI: 0.08, 1.73). For NO_2_, an IQR increase in the instrument was associated with a 0.62% increase in daily deaths (95% CI: –0.12, 1.64). We compared IQR changes for the instrumental variables to have some basis for comparing effects between the models for PM_2.5_, BC, and NO_2_. When the mortality analysis was restricted to days when PM_2.5_ was < 30 μg/m^3^ (which excluded 39 days), we found a 0.84% increase in daily deaths for the same increase in the instrument (95% CI: 0.19, 1.50).

When we used the Granger causality approach, the estimated effect of an IQR change in the instrument for PM_2.5_ remained the same (0.90%; 95% CI: 0.25, 1.96), whereas the forward lagged instrument was not associated with mortality (0.18%; 95% CI: –0.45, 0.81), suggesting no omitted confounders. Although the power for a Granger causality test may not be strong, the much smaller effect size as well as lack of significance both indicate a lack of confounding.

Finally, when we added the mean of PM_2.5_ on lags 0 and 1 to the model in addition to the instrumental variable, the instrumental variable was far from significant (*p* > 0.29) while the PM_2.5_ variable was significant. This indicates that there was no path from instrument to the outcome except through PM_2.5_, and hence that the instrumental variable assumption was valid.

## Discussion

Using a framework based on potential outcomes, we have estimated the causal effect of an IQR increase in local air pollution on daily deaths in Boston. The increase in deaths for an IQR increase in the instrument for exposure was about 0.90% using either particle measure to calibrate the instrument; for NO_2_ it was lower (0.62%) with confidence intervals that crossed zero. Using the approach of Granger causality, we saw no change in the estimated effect of our instrument when controlling for exposure on future days and the association with future exposure was close to zero and far from significant. Further, the association persisted when restricted to days well below the recently tightened U.S. EPA 24-hr standard for PM_2.5_ (35 μg/m^3^), and in a city that never violated the hourly NO_2_ National Ambient Air Quality standard during the study period. Hence, these effects are evident at levels below currently permissible limits.

A key advantage of the instrumental variable approach is that it provides protection against unmeasured confounders. We have approached this in three ways. First, we have shown that if we have a valid instrument, then the association will be causal even in the presence of unmeasured confounders. We focused on the variation in local pollution within deciles of temperature and also stratified on each month of each year. We then chose as instruments variables (PBL height and wind speed) we believed, based on external knowledge, are unlikely to be associated with mortality except through air pollution. Second, we have confirmed that values of the instrument following the day of death are not significantly associated (*p* = 0.57) with daily deaths, and that control for them did not change the estimated effect of the instrument. This assures that omitted confounders with the same broad temporal variability are not confounding our instrument. And third, we have tested the instrument assumption (that the association of the instrument is only through air pollution) by controlling for air pollution, and showing that no significant association with the instrument remained (*p* > 0.29). We believe that this makes a strong case for a causal effect.

Support for this causal interpretation also comes from an extensive toxicological and human exposure literature on some of these local pollutants. For example, Furuyama et al. (2006) found increased oxidative stress in endothelial cells exposed to diesel exhaust, and in humans Rossner et al. (2007) reported increased levels of F-2 isoprostane and 8-OHdG (8-hydroxy-2′-deoxyguanosine) in bus drivers compared with controls. The human study contrasted urinary 8-OHdG in 50 bus drivers and 50 controls measured in three successive seasons in Prague. In logistic regression analysis, PM_2.5_, but not volatile organic compound or polycyclic aromatic hydrocarbon exposure, was associated with 8-OHdG. Romieu et al. (2008) measured malondialdehyde in exhaled breath condensate at 480 visits in a panel of 108 children with asthma seen every 2 weeks, and found it was positively associated with PM_2.5_ at the nearest monitoring station within 5 km of their home and school.

Increased atherosclerosis has also been reported in animals with long-term exposure to particles, much of which was from traffic (Sun et al. 2005, 2008). Another study (Soares et al. 2009) placed hyperlipemic mice in two exposure chambers 20 m from a road. One chamber was filtered to remove particles and the other was not. After 120 days of exposure they documented increased oxidation of low-density lipoprotein, increased thickness of the arterial wall, and greater plaque growth and instability (Soares et al. 2009). Along with the increased oxidative stress, atherosclerosis, and plaque instability, increased thrombosis has also been associated with local pollution. Nemmar et al. (2002, 2003) found that both diesel and ultrafine particles were associated with increased thrombosis in an animal model, and Carlsten et al. (2007, 2008) found that controlled exposure to diesel exhaust increased coagulation markers and thrombosis in human volunteers. Ischemia has likewise been produced experimentally by diesel exposure in a double-blind randomized crossover exposure of 20 people with previous myocardial infarction to 1 hr of dilute diesel exhaust or filtered air (Mills et al. 2007).

An intervention trial in Beijing had 15 young adults (median age, 28 years) walk the streets for 2 hr twice, once wearing a particle-filtering mask, and once without a mask. Blood pressure was measured continuously during the two 2-hr walks and was 7 mmHg lower when wearing the mask (Langrish et al. 2009). These results, combined with the instrumental variable approach and Granger causality model, support a causal interpretation.

The weaker association of the instrumental variables when calibrated to NO_2_ than to particles suggests that local particles may be more important in this relationship, but no definite conclusions can be drawn.

To put this result in context, the mean PM_2.5_, NO_2_, and BC (9.8 μg/m^3^, 18.4 ppb, and 0.7 μg/m^3^) were all greater than their IQRs (6.32 μg/m^3^, 8.4 ppb, and 0.50 μg/m^3^, respectively), indicating that IQR changes in the pollutant concentrations would result in levels above zero, and hence are plausible. Computing the attributable risk for an IQR change in exposure to the instrument, we estimated that local air pollution was responsible for 1,826 deaths in the Boston metropolitan area during the study period. This is a substantial public health burden.

Local air pollution in Boston has multiple sources, including traffic, combustion of fuel oil and residual oil for heating, and wood burning (Masri et al. 2015). Traffic pollution has fallen because of reduced U.S. EPA emission standards on vehicles, low-sulfur diesel oil requirements, the retrofit of particle filters onto buses, and the introduction of compressed natural gas buses for part of the fleet (Masri et al. 2015; U.S. EPA 2012). Continuing retirement of older vehicles will likely continue this trend. Wood burning, on the other hand has increased and now accounts for 19% of particles in Boston (Masri et al. 2015), and though the U.S. EPA has proposed new emission standards for future stoves and furnaces, there is no retrofit requirement. Heating oil, while similar to diesel oil, is still allowed much higher sulfur content. Hence, there are opportunities for local action to reduce this public health burden.

There are several limitations to our study. First, we have assumed we have a valid instrument. Although we have good evidence that this is the case, one can never guarantee it. It is possible that behavior is modified on low-PBL or low–wind speed days in a way that affects mortality risk. A second limitation is that we have provided our proof that an instrumental variable protects against unmeasured confounding in the context of a log-linear model between mortality and air pollution, and assume that model is correct. This is the traditional approach for daily death counts, but we cannot be sure it is correct. In addition, all-cause mortality includes some causes of death unlikely to be associated with air pollution. This decreases power in our analysis, but still leaves us with a valid estimate of the impact on all deaths. The air pollutants, PBL, and wind speed were measured at only one location, which may introduce some error into the instrumental variable, which, if the instrument assumption is valid, should result in an underestimate of risk. Power is always an issue, and the power for a Poisson regression depends on the total number of events. In our case, there were 204,386 deaths during the study period, which indicates good power for our hypothesis tests.

In summary, we have used causal methods to estimate the acute effect of local air pollution on daily deaths, and found that concentrations below current limits are associated with important increases in daily deaths. If, when stratified by month and temperature, our instrument is independent of other causes of mortality, this association is causal, an interpretation supported by toxicological studies.
